# *Trissolcus kozlovi* in North Italy: Host Specificity and Augmentative Releases against *Halyomorpha halys* in Hazelnut Orchards

**DOI:** 10.3390/insects12050464

**Published:** 2021-05-18

**Authors:** Silvia Teresa Moraglio, Francesco Tortorici, Sara Visentin, Marco Giuseppe Pansa, Luciana Tavella

**Affiliations:** Dipartimento di Scienze Agrarie, Forestali e Alimentari (DISAFA), University of Torino, Largo P. Braccini 2, 10095 Grugliasco, Italy; silvia.moraglio@unito.it (S.T.M.); sara.visentin1990@gmail.com (S.V.); marco.pansa@unito.it (M.G.P.); luciana.tavella@unito.it (L.T.)

**Keywords:** brown marmorated stink bug, Pentatomoidea, Scelionidae, egg parasitoid, host range

## Abstract

**Simple Summary:**

The Asian brown marmorated stink bug, *Halyomorpha halys*, is an invasive crop pest introduced into Europe in the 2000s. Due to its high harmfulness, and the increased chemical use for its control in the invaded areas, research has focused on biological control. In North Italy, the native parasitoid *Trissolcus kozlovi* emerged from field-collected *H. halys* eggs and proved to successfully parasitize *H. halys* eggs in the laboratory. Therefore, since little is known on *T. kozlovi*, this study aimed at assessing its physiological host range on 12 bug species in the laboratory, as well as its potential as a biological control agent of *H. halys* in the field by releases in two hazelnut orchards. In the laboratory, among the tested bug species, only *Nezara viridula* was an unsuitable host. On all others, *T. kozlovi* was able to develop, even if at different levels, suggesting that it is as oligophagous as *Trissolcus japonicus*, with which it shares many similarities. In the field, *T. kozlovi* was found to parasitize *H. halys* eggs, but only immediately after field releases. Therefore, further field surveys are required to assess its favorably environmental conditions and its possible interaction with *T. japonicus*, currently present in Italy.

**Abstract:**

*Trissolcus kozlovi* (Hymenoptera: Scelionidae) emerged from field-laid eggs of *Halyomorpha halys* (Hemiptera: Pentatomidae) in North Italy, and it emerged in significantly higher numbers from fresh *H. halys* eggs compared to other native scelionids. Since few data on *T. kozlovi* are available, its host-specificity and some biological traits were investigated in laboratory tests, and its impact after augmentative releases was evaluated in two hazelnut orchards. Among the 12 tested bug species (Hemiptera: Pentatomidae, Scutelleridae), only *Nezara viridula* was an unsuitable host, while the highest offspring proportions were obtained from *Arma custos*, *Pentatoma rufipes*, and *Peribalus strictus*, followed by *Acrosternum heegeri* and *Palomena prasina*. Furthermore, when reared on *P. strictus*, *T. kozlovi* showed a high longevity as well as a high adaptation to *H. halys* eggs. In both hazelnut orchards, *T. kozlovi* emerged from *H. halys* eggs after field releases, but it was not found in the next two years. The physiological host range of *T. kozlovi* was quite similar to that of *T. japonicus*, and probably *T. kozlovi* has just begun to attack *H. halys* as a new host. This aspect needs to be further investigated, as well as its favorable environmental conditions, its distribution and also its possible interaction with *T. japonicus*, currently present in Italy.

## 1. Introduction

*Halyomorpha halys* (Stål) (Hemiptera: Pentatomidae) arrived from East Asia (China, Taiwan, Japan, and Korea) at North America and Europe in the 1990s and 2000s, respectively. In these new areas, it has become one of the major pests of many crops [1,2,3,4], including pome and stone fruits, maize, and hazelnut [5,6,7,8]. Due to the harmfulness of *H. halys* in the newly invaded areas, and considering that most effective chemicals are broad-spectrum insecticides which are also potentially disruptive to natural enemies and pollinators [9], research soon focused on biological control, both in the native [10] and the invaded areas [11].

In the native area, *H. halys* populations are attacked by a complex of egg parasitoids, mainly belonging to the Scelionidae and Eupelmidae [12]. Within the egg parasitoid guild in Beijing and Hebei provinces (China), *Trissolcus japonicus* (Ashmead) (syn. *T. halyomorphae* Yang [13]) (Hymenoptera: Scelionidae) is the predominant species, showing parasitism rates ranging from 50% to 80% and it is, therefore, considered a promising candidate for biological control of *H. halys* [14,15,16]. In Japan, *Trissolcus mitsukurii* (Ashmead) (Hymenoptera: Scelionidae) is reported to be the main egg parasitoid of *H. halys* [17]. Recently, adventive populations of both Asian parasitoids have been detected in Europe, *T. japonicus* first in Switzerland and then in Italy, and *T. mitsukurii* in Italy [18,19,20,21,22]. In Europe, the introduction of new exotic biocontrol agents must be approved by regulatory agencies [23,24]. Currently, the field release of *T. japonicus* has been authorized in Italy by the Italian Ministry for Environment, Land and Sea Protection and started in the summer of 2020. Meanwhile, research in Europe has been carried out on the indigenous parasitoids able to successfully develop from *H. halys* eggs [11].

Among the native European egg parasitoids, the generalist *Anastatus bifasciatus* (Geoffroy) (Hymenoptera: Eupelmidae) was the most widespread, emerging from both field-laid and sentinel *H. halys* egg masses in Italy and Switzerland [19,21,22,25,26,27] and it was soon considered as a potential biological control agent [28,29]. However, during field surveys in North Italy, the overall impact of *A. bifasciatus* on *H. halys* eggs was less than 20% and did not increase over the subsequent years [19], and an augmentative release strategy applied in a 3-year field study in fruit orchards in Switzerland and Italy did not effectively suppress the pest [30].

Another parasitoid, *Trissolcus kozlovi* Rjachovskij (Hymenoptera: Scelionidae) was found emerging from field-collected *H. halys* eggs in North Italy [19,21], and among native scelionid species tested in the laboratory, it was the only one significantly producing offspring from fresh *H. halys* eggs [31]. Other native European egg parasitoids in the genera *Trissolcus* and *Telenomus* (Hymenoptera: Scelionidae) showed in laboratory studies that they were able to oviposit in *H. halys* eggs, and often *Trissolcus* species significantly induced host egg abortion (non-reproductive mortality [32]), but they could not complete development in the exotic host [25,31,33,34,35]. Therefore, *T. kozlovi* seemed to be another potential biological control candidate for *H. halys*, sharing similarity with *T. japonicus*, both morphologically [36] and genetically [19]. However, little information is available about the distribution and host range of this species. In fact, *T. kozlovi* was recorded for the first time in Italy in 2016, emerging from eggs of *H. halys*, *Arma custos* (F.) and a *Carpocoris* species (Hemiptera: Pentatomidae) collected just at two sites, and the only other records of its emergence are from Moldova and Russia from eggs of *Pentatoma rufipes* L. and *Palomena prasina* L. (Hemiptera: Pentatomidae) [19,21].

Therefore, the aim of this study was to evaluate: (i) the physiological host range of *T. kozlovi*, with laboratory parasitism no-choice tests on various pentatomid and scutellerid species; (ii) some biological traits (offspring production, longevity) when reared on different hosts; (iii) its potential as biological control agent of *H. halys,* both in the laboratory and in the field by releases in two hazelnut orchards. Moreover, considering the morphological and genetic similarity between *T. kozlovi* and *T. japonicus*, a more in-depth morphological analysis of numerous specimens reared under the same conditions, and mating tests to assess the reproductive isolation were performed to separate these two species.

## 2. Materials and Methods

### 2.1. Insect Collection and Rearing

*Trissolcus kozlovi* was originally obtained from *H. halys* eggs collected in Cavour, North Italy (44°46′52.4″ N 7°22′59.2″ E, 293 m asl) in 2017 [19]. A laboratory colony was maintained by providing mated females with fresh and frozen (at −20 °C for at least 24 h) *H. halys* egg masses. Rearing was conducted in plastic containers (10 cm diameter, 5 cm height) with honey and wet cotton as a food source, at 24 ± 1 °C, 65 ± 5% RH and 16:8 h L:D.

Almost all bug species (Hemiptera: Pentatomidae, Scutelleridae), including *H. halys*, were collected in Piedmont, North Italy, from March to September in 2018–2019. During field surveys, individuals were collected by visual inspection or plant beating on hedges, wild herbaceous, shrubby and arboreous plants, on cultivated wheat and hazelnut, as previously described [4,19,31]. Only the *Acrosternum heegeri* Fieber colony was already established at DISAFA, started from adults collected in Samegrelo, West Georgia, in autumn, 2017 [4].

In the laboratory, bug species were identified using the available keys [37,38,39,40,41]. Then the insects were reared, each species separately, in polyester cages (BugDorm-4090 Insect Rearing Cage 47.5  ×  47.5  ×  47.5 cm, MegaView Science Co., Ltd., Taichung, Taiwan) at 24 ± 1 °C, 65 ± 5% RH and 16:8 h L:D. Mass-reared specimens were supplied with host plant shoots, *Vicia faba* L. (Fabales: Fabaceae) seedlings, unshelled hazelnuts and apples which were periodically replaced. The mass-reared predator *A. custos* was also provided with adults and larvae of *Plodia interpunctella* (Hübner) (Lepidoptera: Pyralidae) as prey. Mass rearing of *Eurygaster maura* (L.), collected on wheat, was performed in plastic boxes (3 L volume) with holes closed with mesh. Wheat ears and wet cotton were supplied and periodically replaced. All mass rearings were checked daily to collect all freshly-laid egg masses.

In total, 11 pentatomid and one scutellerid species were collected, reared, and used as host for *T. kozlovi*, namely: *A. heegeri*, *A. custos*, *Carpocoris mediterraneus* Tamanini, *Do-lycoris baccarum* (L.), *H. halys*, *Nezara viridula* (L.), *P. prasina*, *P. rufipes*, *Peribalus strictus* (F.), *Piezodorus lituratus* (F.), *Rhaphigaster nebulosa* (Poda) (Hemiptera: Pentatomidae), and *E. maura* (Hemiptera: Scutelleridae).

### 2.2. Morphological Analysis of *T. kozlovi*

*Trissolcus kozlovi* specimens were preserved in ethanol; later, some of them were dried and mounted on card points for morphological examination. A Leitz large-field stereo microscope TS with up to 160× magnification and a spotlight Leica CLS 150X, diffused with a semi-transparent light shield, were used for morphological diagnosis. The photographs were taken using a Canon 90D camera equipped with extension tube, 20× LWD microscope lens mounted on a macro-rail and illuminated with two speedlite flashes. The frames were merged with Zerene Stacker (PMax algorithm).

For the morphological identification, the key to Palearctic *Trissolcus* species provided in Talamas et al. [36] was used. Terminology for surface sculpture follows Harris [42], while morphological terminology follows Mikó et al. [43] for the head and mesosoma except for: haol—the line between the dorsal margin of the hyperoccipital and posterior margin of the anterior ocellus; Johnson [44] for the metasoma.

Females of both *T. kozlovi* and *T. japonicus* were compared to perform a character state analysis to definitively distinguish the two sibling species. Specimens of *T. japonicus* were obtained from a laboratory colony started from material provided by Council for Agricultural Research and Economics (CREA) Research Centre for Plant Protection and Certification (Florence, Italy) and maintained as described for *T. kozlovi*.

### 2.3. Mating Tests and Reproductive Isolation between *T. kozlovi* and *T. japonicus*

To start the tests, parasitized *H. halys* egg masses were collected from *T. kozlovi* and *T. japonicus* rearing colonies, and virgin females that emerged after the removal of males were used.

To determine reproductive isolation of *T. kozlovi* from *T. japonicus*, 1–2 day-old virgin females and males were used. The following six combinations were compared: (1) *T. kozlovi* (♀) × *T. japonicus* (♂); (2) *T. japonicus* (♀) × *T. kozlovi* (♂); (3) *T. kozlovi* (♀) × *T. kozlovi* (♂); (4) *T. japonicus* (♀) × *T. japonicus* (♂); (5) only *T. kozlovi* (♀); (6) only *T. japonicus* (♀).

Each combination was replicated seven times. For tests 1 to 4, each pair of wasps was isolated in a glass tube (24 mm diameter, 120 mm length) for 24–36 h to ensure that mating occurred, while for tests 5 and 6, the females were not paired with males. After this period, one *H. halys* egg mass was exposed to each female for 24 h. Then the egg masses were moved to other glass tubes until offspring emergence.

Tests 1 to 4 were considered successful when the emerged offspring included females, while tests 5 and 6 were considered successful when the emerged offspring included only males because it is known that mode of sex determination in *Trissolcus*, as in most Hymenoptera [45], is arrhenotokous parthenogenesis: only mated females can produce female offspring, while unmated females produce male offspring.

### 2.4. Physiological Host Range of *T. kozlovi* Assessed in Laboratory

In no-choice tests, 1–2-week-old *T. kozlovi* naive, mated females were used. Egg masses of all 12 bug species were collected daily from mass rearing and immediately used. As already described [31], each female parasitoid, tested only once, was offered a single egg mass in a glass tube (24 mm diameter, 120 mm length) closed with a cotton plug and fed with honey drops for 24 h. Then the exposed egg masses were removed from the tubes, individually reared in plastic Petri dishes (60 mm diameter), and checked daily until eggs hatched, or adult parasitoids emerged, which were counted and sexed. No-choice tests were carried out in climatic chambers at 24 ± 1 °C, 65 ± 5% RH and 16:8 h L:D. The following parameters were recorded for each egg mass: (i) number of eggs from which bug nymphs emerged; (ii) number of eggs from which *T. kozlovi* adults emerged; (iii) number of unhatched eggs. The sex ratio was calculated as described for *A. bifasciatus* [28], and expressed as the percentage of female *T. kozlovi* offspring for each single egg mass, which was then averaged for each host species.

At the end of the no-choice tests, proportions of females producing offspring, exploitation (mean offspring emergence per parasitized egg mass), and impact (mean offspring emergence per egg mass) were compared among host species using the general linear model (GLM) procedure of the software IBM SPSS^®^ Statistics 25 (IBM Corp., Armonk, NY, USA) with a binomial distribution model and a logit link function. Means were then separated at *p*  <  0.05 using the Bonferroni test.

### 2.5. Offspring Production and Longevity of *T. kozlovi* Emerged from Different Hosts

Offspring production on *H. halys* eggs and longevity of *T. kozlovi* emerged from the tested host species were determined as described for *A. bifasciatus* [28]. All the experiments were carried out in climatic chambers at 24 ± 1 °C, 65 ± 5% RH and 16:8 h L:D.

To evaluate the offspring production, 1–2-week-old *T. kozlovi* females (no. 19–36) that emerged from the different hosts were used. Each parasitoid female was offered a single fresh *H. halys* egg mass in a glass tube for 24 h, as described for no-choice tests. At the end of the experiments, mean proportions of parasitized eggs within each egg mass were compared among host species using the GLM procedure of the software IBM SPSS^®^ Statistics 25 with a binomial distribution model and a logit link function. Means were then separated at *p*  <  0.05 using the Bonferroni test under the GLM procedure.

To assess longevity, all females and males emerging from the same egg mass were kept in a glass tube, closed with a cotton plug, and fed with honey drops on cardboard. Females used for the offspring production experiment were singly kept in glass tubes at the same conditions. Adult mortality was daily recorded and dead parasitoids removed. Longevity of females (naive or previously exposed to egg mass) and males emerging from different host species was compared with a stratified log-rank test using the software IBM SPSS^®^ Statistics 25.

### 2.6. Field Releases of *T. kozlovi*

Releases were conducted during 2018 in Piedmont, North Italy in two hazelnut orchards where no insecticides had been applied (Table 1, Figure S1). In mid-July an aggregation pheromone dispenser (Pherocon^®^ BMSB dual lure, Trécé Inc., Adair, OK, USA) was placed on the central plant of a border row, in order to attract more bugs and increase oviposition (Figure S1). Two weeks later, all hazelnut trees of both orchards were inspected to find and collect all *H. halys* egg masses to verify the parasitism before the release of *T. kozlovi*. The release started three days later and was repeated three times at 2-week intervals. At each release, 400 females and 100 males of *T. kozlovi* were distributed on the tree baited with the pheromone lure. During each release, at least 25 sentinel egg masses, 50 if available, were exposed (2–3 per tree if 25, 4–5 per tree if 50) on the border row where the pheromone dispenser was placed, and on the orthogonal central row. Meanwhile, all hazelnut trees of the orchard were inspected to check for the presence of field-laid egg masses of *H. halys*, which were labeled (Table 1, Figure S1). Two days after each release, all egg masses, exposed and labeled, were collected and a further 25 or 50 sentinel ones were exposed, while any new, field-laid ones were labeled. After another two days, all egg masses, exposed or labeled, were collected. The sentinel egg masses were glued on a white tag and included both fresh and frozen eggs: on the day of each exposure, all fresh egg masses available from mass rearing were collected, and if there was not enough, frozen ones (fresh egg masses kept at −20 °C) were added (Table 1).

All collected egg masses, sentinel or field-laid, were singly reared at the same conditions as in the laboratory tests, until all eggs hatched or adult parasitoids emerged. The adult parasitoids were examined and separated according to their taxa and sexed. All parasitoids were then stored in 99% ethanol prior to identification. At the end of the season, all collected egg masses were inspected under a Leica stereo microscope S6D with a magnification up to 40 × to assess the fate of all eggs. Egg fate categories were assigned to individual eggs within each egg mass [31]: (1) hatched, where *H. halys* emerged from the vacated egg; (2) parasitized, where parasitoid emergence had occurred; (3) sucked, where the egg was empty and one or more stylet sheaths protruded from the egg; (4) broken, where egg was empty and the chorion was broken in at least one place; (5) unhatched, where a direct cause of mortality could not be properly diagnosed. Additionally, parasitized eggs from which parasitoids had emerged were ascribed to a parasitoid family [14,31,46].

In the next two years, 2019 and 2020, a monthly survey was conducted in both hazelnut orchards from June to August. During the survey, all pentatomid egg masses found by 1-h visual inspection on trees were collected and reared in laboratory as explained above. All emerged parasitoids were counted and identified.

## 3. Results

### 3.1. Morphological Analysis of *T. kozlovi*

**Figure 1:**
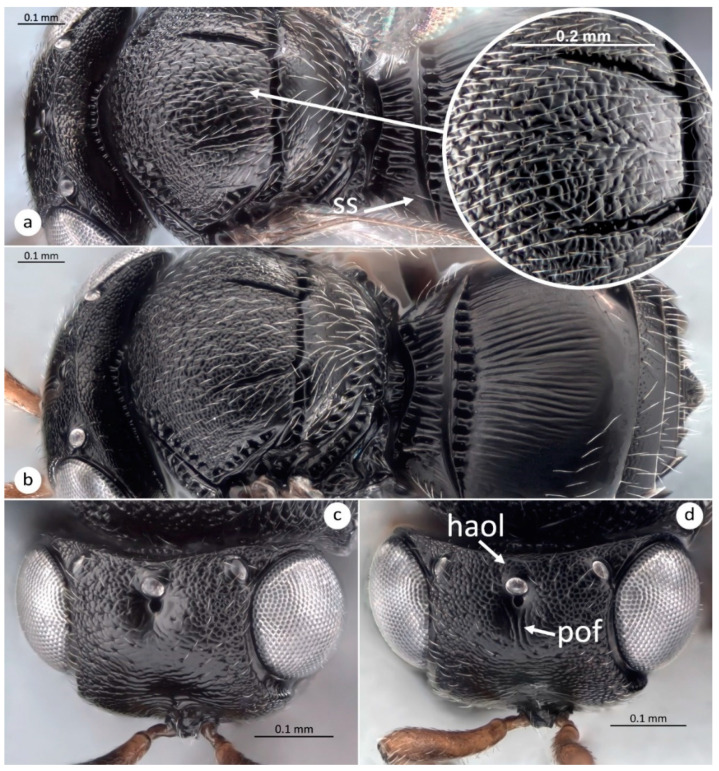
*Trissolcus kozlovi* and *T. japonicus*. (**a**)—female of *T.* *kozlovi*, head, mesosoma (in detail median area of the mesoscutum), metasoma (**ss** = sublateral seta), dorsal view; (**b**)—female of *T.* *japonicus*, head, mesosoma, metasoma, dorsal view; (**c**)—female of *T.* *kozlovi*, head, anterodorsal view; (**d**)—female of *T.* *japonicus*, head, anterodorsal view (**haol** = line between the dorsal margin of hyperoccipital carina and posterior margin of anterior ocellus; **pof** = preocellar furrow).

From the comparison of *T. kozlovi* and *T. japonicus*, the sculpture on the mesoscutum is finely colliculate anteriorly and coarsely colliculate with obliquely oriented sculpture posteriorly on the median area between the notauli in *T. kozlovi* (Figure 1a), while in *T. japonicus* the finely colliculate sculpture on the mesoscutum is uniformly extensive, reaching the posterior margin between the notauli (Figure 1b). Another useful diagnostic character is one sublateral seta (ss) on each side of tergite 1 (T1), always present in *T. kozlovi* (Figure 1a) and almost always absent in *T. japonicus* (Figure 1b). This character was recorded as an additional character to separate *T. japonicus* from *Trissolcus plautiae* (Watanabe) [47]. Rarely a sublateral seta is present on one side of T1 in some specimens of *T. japonicus*, and even more rarely, the setae are present on T1 in all the specimens emerging from the same egg mass. The sublateral setae are always absent in specimens of *T. japonicus* obtained under the same controlled rearing conditions. The combination of the presence of longitudinal rugae below the anterior ocellus (preocellar furrow in Sabbatini Peverieri et al. [20]) and microsculpture finely colliculate along the line between the dorsal margin of the hyperoccipital carina and the posterior margin of the anterior ocellus in *T. japonicus* (Figure 1d) is absent in *T. kozlovi* (Figure 1c).

### 3.2. Mating Tests and Reproductive Isolation *between T. kozlovi and T. japonicus*

**Figure 2:**
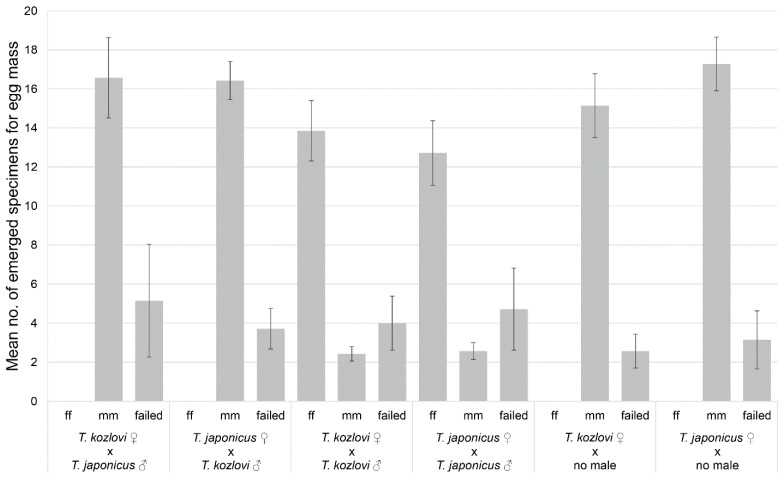
Mean numbers (± S.D.) of emerged females (ff) and males (mm) and unhatched eggs (failed) in each egg mass for the combinations: (1) *T. kozlovi* ♀ × *T. japonicus* ♂; (2) *T. japonicus* ♀× *T. kozlovi* ♂; (3) *T. kozlovi* ♀ × ♂; (4) *T. japonicus* ♀ × ♂; (5) only *T. kozlovi* ♀; (6) only *T. japonicus* ♀.

Females used for intraspecific combinations *T. kozlovi* (♀ × ♂, test 3) and *T. japonicus* (♀ × ♀, test 4) successfully produced female offspring (Figure 2) with female to male ratios of 5.7:1 and of 4.9:1, respectively.

All females used for interspecific combinations *T. kozlovi* ♀ × *T. japonicus* ♀ (test 1) and *T. japonicus* ♀ × *T. kozlovi* ♀ (test 2) produced exclusively male offspring, as expected, as well as the combinations only *T. kozlovi* ♀ (test 5) and only *T. japonicus* ♀ (test 6) (Figure 2).

### 3.3. Physiological Host Range of *T. kozlovi* Assessed in Laboratory

Except *N. viridula*, which was found to be an unsuitable host for *T. kozlovi* as no parasitoids emerged from the exposed egg masses, all the other 11 species were suitable for the development *of T. kozlovi*, but at significantly different levels (Table 2). Proportions of *T. kozlovi* females producing offspring were significantly higher in *A. heegeri*, *A. custos*, *P. prasina*, *P. rufipes*, and *P. strictus*, and lower in *H. halys*, *P. lituratus* and the scutellerid *E. maura*. The mean efficiency (offspring per egg mass with at least one parasitoid emergence) was significantly higher in *P. rufipes* and *P. strictus*, and lower in *H. halys*. Consequently, the mean total impact (mean offspring emergence per egg mass) was significantly higher in *P. rufipes* and *P. strictus* (over 79%), and lower in *P. lituratus*, *H. halys*, and *E. maura* (lower than 20%). The impact was from 30% to 38% in *C. mediterraneus*, *D. baccarum*, and *R. nebulosa*, while from 68% to 80% in the rest of the species.

### 3.4. Offspring Production and Longevity of *T. kozlovi* Emerged from Different Hosts

Offspring efficiency of *T. kozlovi* females on *H. halys* eggs was affected by the host species from which they had emerged (Table 3). A higher proportion of *T. kozlovi* females produced offspring on *H. halys* eggs when reared from *P. rufipes* rather than from *A. custos* and *P. prasina*. The mean efficiency was significantly higher for females emerged from *A. heegeri*, *D. baccarum*, and *E. maura* than for females emerged from *A. custos* and *H. halys*. Consequently, the total impact on *H. halys*, ranging from 2% to 13%, was higher for females reared from *P. rufipes* than for females reared from *A. custos*, *C. mediterraneus*, *H. halys*, and *P. prasina*.

The longevity of both *T. kozlovi* females and males was affected by the host species (Table 4). Longevity of females ranged from 8–30 days to more than 130 days when they had emerged from *H. halys* and *P. rufipes*, and from *E. maura*, respectively. Longevity of males ranged from 8–10 days to 78 days when they had emerged from *A. custos* and *P. rufipes*, and from *E. maura*, respectively. Longevity of males was generally lower than that of females.

### 3.5. Field Releases of *T. kozlovi*

In both hazelnut orchards, *T. kozlovi* was not found to emerge from field-laid eggs of *H. halys* before the releases, while after the first release it emerged from 5.1% and 8.1% of the field-laid egg masses in site 1 and 2, respectively, and after the third release from 3.7% and 5.6% of the field-laid egg masses in site 1 and 2, respectively (Table 5). *Trissolcus kozlovi* was never collected from sentinel eggs in site 2, neither fresh nor frozen, while in site 1 it was found to emerge from 9.1% of frozen sentinel egg masses after the first release, and from 2.6% and 6.7% of fresh sentinel egg masses after the second and the third release, respectively (Table 5). The overall impact of *T. kozlovi* on *H. halys* eggs, field-laid or sentinel, was lower than 3% throughout the season. *Anastatus bifasciatus* was the only other parasitoid that emerged from *H. halys* eggs, and it was found in both orchards already before the first release of *T. kozlovi* (Table 5). In site 2, *A. bifasciatus* emerged only from field-laid *H. halys* eggs, and only in two periods, while in site 1 it emerged from both field-laid and fresh sentinel *H. halys* egg masses in all periods, while only in two of the three periods after *T. kozlovi* releases from frozen sentinel *H. halys* egg masses (Table 5).

In the field surveys conducted in the next two years, *T. kozlovi* was not found emerging from 107 and 49 *H. halys* egg masses which were collected in site 1 and in site 2, respectively (data not shown). Furthermore, *T. kozlovi* was never found emerging from 80 and 12 egg masses of other pentatomid species (i.e., *A. custos*, *D. baccarum*, *Eurydema* spp., *G. lineatum*, *N. viridula*, *P. prasina*, *Peribalus* spp., *R. nebulosa*), which were collected in site 1 and 2, respectively (data not shown).

## 4. Discussion

The diagnosis reported in Talamas et al. [36] to distinguish *T. kozlovi* from *T. japonicus*, based on the analysis of a few available specimens, is here updated. Here, the analysis was performed on a large number of field-collected and laboratory-reared specimens of both *T. kozlovi* and *T. japonicus*. Therefore, the sculpture of the mesoscutum between the notauli remains the most concrete feature to distinguish the two species. However, from the comparison of a large number of specimens of the two species reared under the same conditions, a list of features have arisen to strengthen the character states useful to distinguish the two species. In the absence of controlled rearing conditions, the variation in character expression reported here should likely be considered as individual variation.

Based on the recent record of *T. japonicus* in the Western Palearctic Region [18] and considering the relative morphological similarity of *T. kozlovi* and *T. japonicus*, the difficulty of resolving a set of discriminatory characters led to mating tests to support morphological analyses. Therefore, the observation of morphological differences in specimens reared under the same conditions, related to molecular [19] and biological data, accurately reflects that *T. kozlovi* and *T. japonicus* are two separate species.

The distribution, biology and host range of *T. kozlovi* were largely unknown. Therefore, this study has contributed to the knowledge of the physiological host range of *T. kozlovi* with respect to 11 pentatomid and one scutellerid species present in North Italy. In addition to morphological and genetic similarity with *T. japonicus*, in this study *T. kozlovi* was also found to have some similarity with the physiological host range shown by *T. japonicus* on some European bug species [4,23]. The most suitable hosts for *T. kozlovi* were indeed *A. custos*, *P. rufipes* and *P. strictus*; on these hosts the highest percentages of parasitized egg masses with offspring production, the highest efficiency, and consequently, the highest impact on the exposed eggs were observed. Similarly, these bug species were suitable hosts for *T. japonicus*, although *P. strictus* showed contrasting results [4,23]. Moreover, *T. kozlovi* successfully parasitized high proportions of egg masses of *A. heegheri* and *P. prasina*, but with a lower efficiency, resulting in a lower impact. Therefore, these two hosts seem to be still suitable, but the low efficiency could be due to the number of eggs per egg mass: in fact, *A. custos*, *P. rufipes* and *P. strictus* lay egg masses consisting of 12–13 eggs on average, while egg masses of *A. heegeri* and *P. prasina* consist of 24–25 eggs on average. This would suggest that *T. kozlovi* is adapted to hosts with a small number of eggs per egg mass and has a lower egg load than *T. japonicus* (and other *Trissolcus* species), which was more efficient on *P. prasina* [4,23].

*Trissolcus kozlovi* was less efficient on egg masses of *C. mediterraneus*, *D. baccarum,* and *R. nebulosa*, despite the low number of eggs per egg mass, therefore these three hosts are less suitable for its development. The same behavior was observed for *T. japonicus* on *C. mediterraneus* and *D. baccarum*, but not for *R. nebulosa* which seemed to be more suitable for the exotic parasitoid [4,23]. Finally, *P. lituratus* and *E. maura* were poorly suitable hosts for *T. kozlovi* and *T. japonicus* [4,23]. No *T. kozlovi* or *T. japonicus* emerged from *N. viridula* eggs [4,23,48]. In this study, *H. halys* was also shown to be a less suitable host for *T. kozlovi*, emerging from *H. halys* eggs in lower numbers than in a previous study [31]. This finding should be further investigated since *T. kozlovi* was the most abundant native scelionid species that emerged from field-collected eggs of the exotic bug [19].

Longevity of *T. kozlovi* progeny from the different hosts was sometimes inversely proportional to its impact: in fact, the longest-lived females and males emerged from *E. maura*, a less suitable host, while those with shorter lives emerged from *A. custos* and *P. rufipes*, two of the most suitable hosts. The adults that emerged from *H. halys* were also not long-lived. In *T. kozlovi*, longevity seemed not to be strictly related to the egg shape or volume, as observed for *T. mitsukurii* [49], since the eggs of *E. maura*, *A. custos* and *P. rufipes* are very similar in both shape and volume. The eggs of *P. strictus* have instead a smaller volume [23] but the longevity of females and males that emerged from them was high, suggesting that a larger egg volume does not always translate into a higher suitability for the progeny.

The impact on *H. halys* eggs of females that emerged from the different hosts did not reflect the host suitability or their longevity. The highest impact was obtained from females that emerged from *P. rufipes*, while the lowest from females that emerged from *A. custos* and *P. prasina.* Overall, the impact of *T. kozlovi* on the eggs of *H. halys* was lower than that observed in a previous study [31]. However, it should be remembered *H. halys* is probably a new host, to which *T. kozlovi* has just begun to attack, considering that the known area where *T. kozlovi* is present has been recently colonized by *H. halys*.

Overall, *T. kozlovi* proved to be oligophagous, as assessed for *T. japonicus* [4,16,23,50], and consistent with what was observed for other *Trissolcus* species [25,31]. Among the tested species, *P. strictus* seemed to be a good host on which *T. kozlovi* had a high impact, and its offspring showed high longevity, as well as high adaptation to *H. halys* eggs. *Arma custos* and *P. rufipes*, followed by *A. heegeri* and *P. prasina*, were highly parasitized in no-choice tests, but the offspring was generally less long-lived. Currently, the physiological host range assessed in the laboratory matches the ecological host range partially observed in the field. Indeed, *T. kozlovi* emerged from the field-collected eggs of *A. custos*, *Carpocoris* sp., *H. halys*, *P. rufipes*, and *P. prasina* [19,21], but so far there are few records. Moreover, little information is available on *P. strictus* parasitoids, as its egg masses were found in the field in North Italy only in one year, and only *Telenomus* species were obtained from them [31]. Further field-collection of egg masses, especially in the area where its presence has been recorded, is required to determine if the physiological host range of *T. kozlovi* matches its ecological host range, and to assess its abundance and distribution.

During field releases, *T. kozlovi* successfully parasitized *H. halys* egg masses, both field-laid and sentinel, and initially gave hope for its establishment in the field and for an increasing impact on *H. halys* eggs. However, its impact did not increase, even after subsequent releases, and it was not found in the next two years. Even in this case, further field surveys will be necessary to assess on which plants and in which environments *T. kozlovi* is naturally present, since in North Italy it was found emerging from egg masses collected on maples [31] and on *Vitis vinifera* L. [21].

## 5. Conclusions

Few data were so far available on *T. kozlovi*, and this study contributed to the knowledge about its occurrence, host range, and biology. Here, *T. kozlovi* and *T. japonicus* are definitively confirmed as two distinct species, despite their close similarity in morphological characters. Their physiological host range is also similar, but *T. kozlovi* proved to be less efficient, probably due to a lower egg load as an adaptation to hosts producing egg masses consisting of fewer eggs. This aspect needs to be further investigated, as well as the favorable environmental conditions, as *T. kozlovi* was found to parasitize *H. halys* eggs in hazelnut orchards, but only immediately after field releases. Therefore, further field surveys are needed to assess its distribution and also its possible interaction with *T. japonicus*, currently present in North Italy.

## Figures and Tables

**Table 1:** Numbers of *Halyomorpha halys* sentinel egg masses (fresh and frozen) exposed after each release of *Trissolcus kozlovi* adults in the two hazelnut orchards in Piedmont, North Italy, in 2018.

Site	Coordinates, Altitude	Release	Date	No. Sentinel Egg Masses		
				Total	Fresh	Frozen
1 Chieri (TO)	5°02′28.2″ N	1st	23 July	100	32	68
	7°50′03.9″ E	2nd	13 August	50	43	7
	335 m asl	3rd	27 August	50	16	34
2 Castellar (CN)	44°37′32.4″ N	1st	30 July	100	21	79
	7°26′37.8″ E	2nd	20 August	50	47	3
	337 m asl	3rd	3 September	50	15	35

**Table 2:** Outcomes of no-choice tests: numbers of exposed egg masses (=no. of tested *Trissolcus kozlovi* females) and mean numbers (±SE) of eggs per exposed egg mass, percentages of females producing offspring, mean percentage (±SE) of offspring emergence within each parasitized egg mass and within each exposed egg mass of the tested bug species, and mean proportion (±SE) of females per parasitized egg mass. In columns, values followed by the same letter are not significantly different (Bonferroni test, *p* < 0.05, under GLM procedure with binomial distribution and logit link).

Species	No. Exposed Egg Masses	No. Eggs per Exposed Egg Mass	% Females Producing Offspring	% Parasitized Eggs per Parasitized Egg Mass	% Parasitized Eggs per Exposed Egg Mass	Sex Ratio (Proportion of Females)
*Acrosternum heegeri*	13	24.54 ± 4.37	84.62 a	79.97 ± 8.18 b c	67.67 ± 10.80 b	90.49 ± 1.75
*Arma custos*	25	12.64 ± 1.23	96.00 a	80.43 ± 5.75 b c	77.22 ± 6.38 ab	78.51 ± 6.72
*Carpocoris mediterraneus*	14	14.57 ± 1.49	57.14 ab	67.17 ± 11.24 c	38.38 ± 11.13 c	81.29 ± 3.37
*Dolycoris baccarum*	21	31.52 ± 2.62	70.00 ab	45.92 ± 7.04 d	30.62 ± 6.70 c	87.57 ± 1.14
*Halyomorpha hlays*	32	27.06 ± 0.31	31.25 bc	17.76 ± 5.21 e	5.55 ± 2.16 e	86.05 ± 4.87
*Nezara viridula*	20	58.55 ± 5.14	0.00 d		0.00 ± 0.00 g	
*Palomena prasina*	34	25.53 ± 1.68	87.50 a	81.03 ± 4.77 c	70.90 ± 6.36 b	72.10 ± 5.78
*Pentatoma rufipes*	25	12.25 ± 0.42	91.67 a	86.84 ± 4.10 ab	79.60 ± 5.53 a	85.30 ± 4.44
*Peribalus strictus*	9	12.56 ± 0.93	88.89 a	89.74 ± 8.28 a	79.77 ± 12.36 a	72.52 ± 9.68
*Piezodorus lituratus*	20	18.60 ± 1.58	5.00 cd	42.86	2.14 ± 2.14 f	66.66
*Rhaphigaster nebulosa*	26	14.65 ± 0.55	65.38 ab	45.96 ± 8.24 d	30.05 ± 6.90 c	87.97 ± 5.08
*Eurygaster maura*	21	13.43 ± 0.21	23.81 bcd	71.76 ± 9.33 bc	17.09 ± 7.13 d	89.56 ± 2.58
	GLM	Wald χ^2^	62.761	452.656	1045.535	
		*df*	10	9	10	
		*p*	<0.001	<0.001	<0.001	

**Table 3:** Offspring production on *Halyomorpha halys* eggs of *Trissolcus kozlovi* females emerged from different host species: percentage of females producing offspring, mean percentage (±SE) of offspring emergence within each parasitized egg mass and within each exposed egg mass. In column, values followed by the same letter are not significantly different (Bonferroni test, *p* < 0.05, under GLM procedure with binomial distribution and logit link).

Host Species	No. Females	% Females Producing Offspring	% Parasitized Eggs per Parasitized Egg Mass	% Parasitized Eggs per Exposed Egg Mass
*Acrosternum heegeri*	20	35.00 ab	26.23 ± 9.51 a	9.18 ± 4.27 ab
*Arma custos*	30	33.33 b	6.29 ± 1.30 d	2.10 ± 0.69 c
*Carpocoris mediterraneus*	20	40.00 ab	12.42 ± 3.78 bc d	4.97 ± 2.01 bc
*Dolycoris baccarum*	20	45.00 ab	19.91 ± 5.30 ab	8.96 ± 3.24 ab
*Halyomorpha halys*	28	42.86 ab	10.27 ± 2.36 cd	4.56 ± 1.40 bc
*Palomena prasina*	21	23.81 b	12.41 ± 3.87 a bcd	2.96 ± 1.45 c
*Pentatoma rufipes*	36	72.22 a	18.14 ± 3.54 abc	13.10 ± 2.89 a
*Peribalus strictus*	20	50.00 ab	10.17 ± 9.05 abc	7.97 ± 4.77 ab
*Rhaphigaster nebulosa*	19	63.16 ab	17.49 ± 3.39 abc	11.05 ± 2.90 ab
*Eurygaster maura*	20	40.00 ab	21.13 ± 8.84 ab	8.45 ± 4.14 ab
GLM	Wald χ^2^	18.644	53.826	99.242
	*df*	9	9	9
	*p*	0.028	<0.001	<0.001

**Table 4:** Mean longevity of *Trissolcus kozlovi* females previously exposed to egg mass, naive females, and males, reared from 12 Pentatomidae and Scutelleridae species. In each column, values followed by the same letter are not significantly different (stratified log-rank test).

Host Species	No. Females Exposed to Eggs	Mean Longevity (Days)	No. Naïve Females	Mean Longevity (Days)	No. Males	Mean Longevity (Days)
*Acrosternum heegeri*	19	71.58 ± 6.89 de	56	79.29 ± 4.62 bc	11	32.82 ± 8.62 bc
*Arma custos*	8	35.50 ± 15.62 def	15	90.20 ± 8.13 bc	18	8.22 ± 2.81 d
*Carpocoris mediterraneus*	16	96.25 ± 6.03 ab	24	78.04 ± 7.41 bcd	9	36.11 ± 9.40 bc
*Dolycoris baccarum*	18	60.56 ± 8.15 e	100	65.99 ± 3.40 e	22	19.27 ± 3.57 c
*Halyomorpha halys*	26	15.65 ± 4.23 f	81	29.95 ± 4.53 f	65	29.12 ± 2.54 b
*Nezara viridula*	-				-	
*Palomena prasina*	22	88.73 ± 3.54 cd	59	70.58 ± 5.48 de	140	26.24 ± 1.50 bc
*Pentatoma rufipes*	27	17.85 ± 2.72 f	23	8.48 ± 0.34 g	10	9.60 ± 1.01 d
*Peribalus strictus*	20	100.35 ± 4.51 ab	45	78.73 ± 4.47 cde	23	33.91 ± 4.57 b
*Piezodorus lituratus*	-		2	100.00 ± 9.00	1	54.00
*Rhaphigaster nebulosa*	17	98.12 ± 2.80 bc	45	94.98 ± 3.60 b	29	32.45 ± 4.72 b
*Eurygaster maura*	19	105.32 ± 4.77 a	13	137.85 ± 6.64 a	6	78.50 ± 12.71 a
GLM	Wald χ^2^	207.250		231.531		89.347
	*df*	9		10		9
	*p*	<0.001		<0.001		<0.001

**Table 5:** Numbers of *Halyomorpha halys* egg masses (and eggs), field-laid and sentinel (fresh and frozen) egg masses, and numbers of parasitized ones by *Trissolcus kozlovi* (***Tk***) or *Anastatus bifasciatus* (***Ab***), before and after releasing *T. kozlovi* in two hazelnut orchards in Piedmont, North Italy, in 2018.

Site	Period	No. Egg Masses (No. of Eggs)								
		Field-Laid	Parasitized		Sentinel Fresh	Parasitized		Sentinel Frozen	Parasitized	
			*Tk*	*Ab*		*Tk*	*Ab*		*Tk*	*Ab*
1	Pre-release	15 (396)	0	4 (49)						
	1st release	59 (1543)	3 (4)	4 (35)	22 (528)	0	2 (32)	50 (839)	2 (13)	2 (19)
	2nd release	65 (1753)	0	6 (62)	38 (950)	1 (4)	3 (10)	6 (166)	0	0
	3rd release	81 (2075)	3 (22)	12 (128)	15 (302)	1 (1)	2 (4)	29 (770)	0	1 (1)
2	Pre-release	10 (224)	0	1 (10)						
	1st release	37 (978)	3 (27)	2 (13)	19 (431)	0	0	74 (1907)	0	0
	2nd release	91 (2411)	0	3 (30)	47 (926)	0	0	3 (85)	0	0
	3rd release	18 (460)	1 (1)	0	3 (65)	0	0	9 (154)	0	0

## Supplementary Materials

The following are available online at https://www.mdpi.com/article/10.3390/insects12050464/s1, Figure S1: Hazelnut orchards where *Trissolcus kozlovi* was released in 2018.
