# Supplementary figures and images for: Trissolcus kozlovi in North Italy: Host Specificity and Augmentative Releases against Halyomorpha halys in Hazelnut Orchards
Source: Insects. 2021 May 18;12(5):464. doi: 10.3390/insects12050464 (PMC8157288; doi:10.3390/insects12050464)

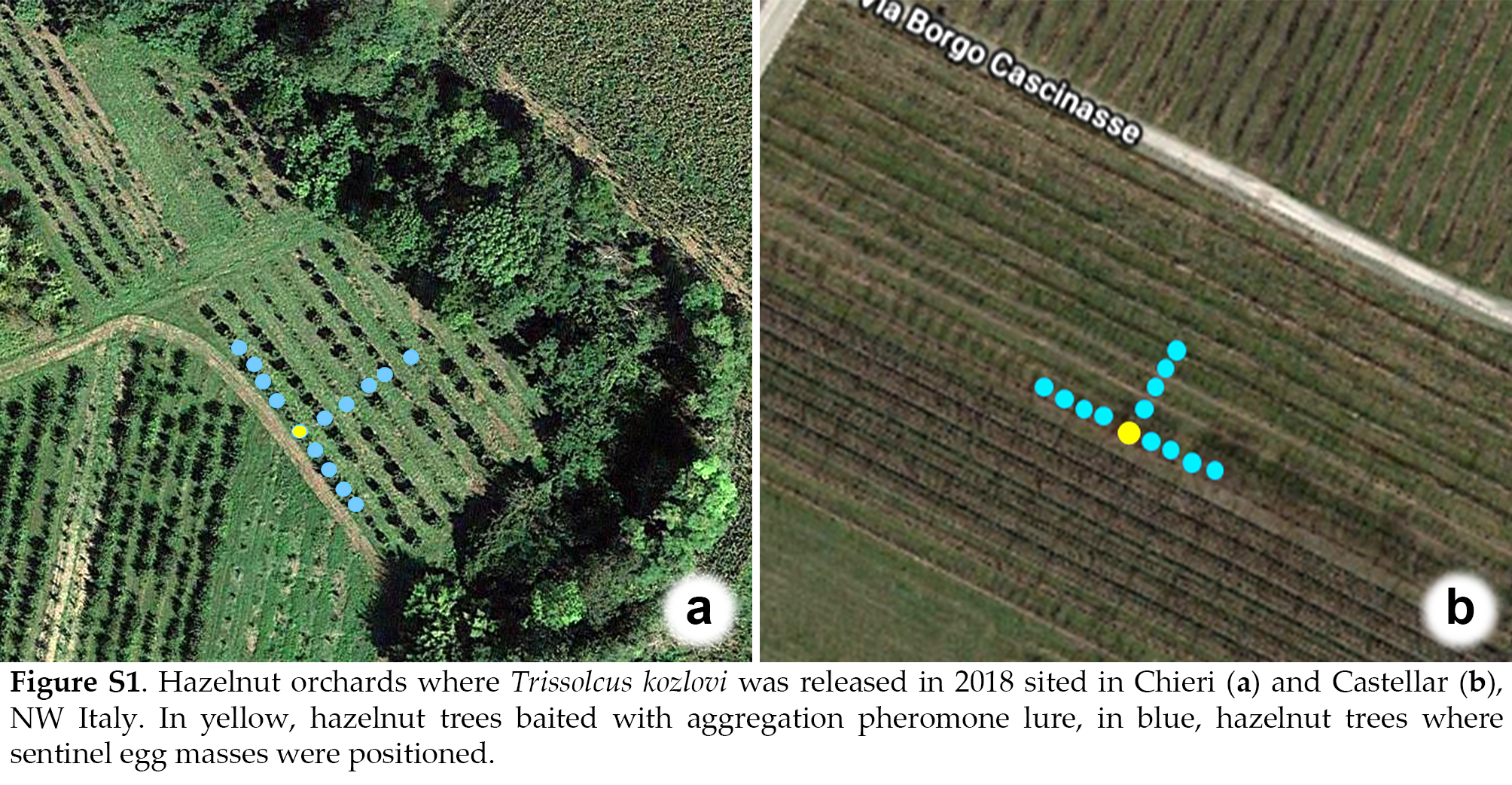

Supplement: Supplementary file 1 [file insects-12-00464-s001.zip › insects-1184585-supplementary.tif]
